# Scanning electron microscopy, biochemical and enzymatic studies to evaluate hydro-priming and cold plasma treatment effects on the germination of *Salvia leriifolia* Benth. seeds

**DOI:** 10.3389/fpls.2022.1035296

**Published:** 2023-01-20

**Authors:** Seyedeh Parisa Ghodsimaab, Hassan Makarian, Ziba Ghasimi Hagh, Manoochehr Gholipoor

**Affiliations:** ^1^ Department of Agronomy and Plant Breeding, Faculty of Agriculture, Shahrood University of Technology, Shahrood, Iran; ^2^ Department of Horticulture Science and Plant Protection, Faculty of Agriculture, Shahrood University of Technology, Shahrood, Iran

**Keywords:** cold plasma, *S. leriifolia* (Noroozak), SEM, uncovered seed, antioxidant and hydrolytic enzymes

## Abstract

Finding a suitable method to increase seed germination rates of medicinal plants is critical to saving them from extinction. The effects of cold plasma (CP) treatments (using surface power densities of 80 and 100 W, with exposure times of 0, 120, 180, and 240 s) and incorporating hydropriming (carried out for 24 and 2 h on normal and uncovered seeds, respectively) to enhance the seed germination of *Salvia leriifolia* Benth a native endangered Iranian medicinal plant, were evaluated in this study. Scanning electron microscopy (SEM) images identified more destroyed mesh-like structures in hydro-primed and uncovered seeds than in normal and dry seeds. In comparison to the control, and other treatments, employing 100 W of CP for 240 s produced the maximum germination percentage and rate, as well as a seedling vigor of I and II in hydro-primed and uncovered seeds. The levels of α-amylase activity increased when the power and exposure times of CP were increased. The uncovering and hydropriming of *S. leriifolia* seeds resulted in increased enzyme activity. Malondialdehyde (MDA) and hydrogen peroxide (H_2_O_2_) contents were enhanced by increasing the power and exposure time of CP, especially in uncovered and hydro-primed seeds. The activity of antioxidant enzymes, including catalase (CAT) and superoxide dismutase (SOD), was correlated to changes in MDA and H_2_O_2_ levels. Finally, direct contact of CP with uncovered seeds in a short exposure time can improve the germination of *S. leriifolia* seeds *via* microscopic etching and activation of enzymes.

## 1 Introduction

Among 17 native Iranian species of *Salvia*, *S. leriifolia* (Noroozak) Benth. is an endangered perennial medicinal plant that is found in warm regions of Iran, including the Khorasan and Semnan provinces (Rechinger, 1982), where locals tend to eat the roasted seeds of this plant as nuts (Amini et al., 2018). The leaves, roots, and flowers of this medicinal plant contain beneficial secondary metabolites such as caffeic acid, rosmarinic acid, and salvianolic acid B (Modarres et al., 2014). Therefore, most studies on this plant species in recent years have reported its valuable pharmaceutical effects, such as its capacity to lower blood sugar levels, reduce morphine dependence, and act as an anticonvulsant, analgesic, anti-inflammatory, and anti-ulcer treatment (Savelev et al., 2004; Loizzo et al., 2010). A study by Serry et al. (2012) showed that the seeds of *S. leriifolia* are physically and physiologically dormant, so their germination in natural conditions is very low and in field conditions is about 28% (Pour et al., 2010). Seed dormancy, a complex phenomenon, is influenced by both endogenous and external factors. It can result in non-uniform germination and cause difficulty in maintaining plant populations (Shelar et al., 2022). Accelerating germination as a vital process in plant development is essential for the survival of such a species (Gilani et al., 2021). Some technologies, such as scarification, leaching seed, removal of seed coverings or un-coating, red light, darkness, cold storage (short, moderate, and long period), cold stratifications, hydropriming, nanotechnology, and recently, cold plasma (CP), were used on the seeds of different plant species to overcome physical and physiological dormancy, and increase germination (Geneve, 2003; Ji et al., 2016; Hatami et al., 2017; Pang et al., 2021). CP treatment leads to improved seed germination and increased initial growth of plants, and boosts food-packaging characteristics (Sera et al., 2012; Saberi et al., 2018; Priatama et al., 2022; Le et al., 2022). CP contains low-temperature particles, such as neutral molecules, atomic species, and relatively high-temperature electrons. It involves dry chemistry and does not produce contaminated residues (Dhayal et al., 2006), and, so does not affect sensitive materials that come into contact with it (Ling et al., 2015; Thirumdas et al., 2017). Earlier studies have reported that this technology affects the seed surface, resulting in the transmission of oxygen or water through the seed coat (Bormashenko et al., 2012). These events on the surface of seeds increases water uptake and stimulates the digestion of stored compounds that result in a significant increase in seed germination (Hoppanová et al., 2020; Filatova et al., 2020; Sajib et al., 2020). The scanning electron microscopy (SEM) images of mung bean seeds showed CP-induced cracks on the seed surface and that the water contact angle was decreased from 73° in untreated seeds to 0° in CP-treated seeds (Le et al., 2022). In addition to physical changes, the endogenous hormone balance changed, based on the time of CP exposure, as well as the ratio of different reactive species to CP. The high percentage of nitric oxide (NO) in CP particles significantly enhances the activity of gibberellic acid (GA_3_) hormone in seeds (Le et al., 2022). The activity of some enzymes, such as superoxide dismutase (SOD), catalase (CAT), and peroxidase (POD), is increased in plants treated with CP, and the level of lipid peroxidation (MDA) is decreased. To study related to seed’s decontamination with plasma activated water suggested that it has disinfecting ability on the seeds (Machado-Moreira et al., 2021). Air is commonly used as an edible gas to produce plasma by way of radiofrequency (RF) discharge depletion. Previous studies have shown that the release of RF into the air increases the seed strength of wheat, corn, and mung bean (Bormashenko et al., 2012; Filatova et al., 2014; Sadhu et al., 2017). RF treatment reduced the initial germination time to 50% in conventional beans but did not show a significant change in the final germination percentage (Bormashenko et al., 2015).

Seed priming is a simple and environmentally friendly technique for enhancing seed germination efficiency and plant growth (Zulfiqar, 2021; Priatama et al., 2022). This pre-planting technique can improve radicle emergence, germination speed, and seedling establishment by changing the metabolic activity of seeds (Ghassemi-Golezani et al., 2016; Damalas et al., 2019; Zulfiqar et al., 2022). The combination of CP with hydropriming resulted in smoothing of the seed surface (Wang et al., 2017), the regulation of osmotic salts, and the alteration of the subsequent process of water uptake (Lee et al., 2018).

For domestication and breeding of the *S. leriifolia* species, the first step is to find an appropriate solution to ensuring a high percentage of germination. According to the results of Serry et al. (2012) removing the hard and impermeable seed cover of *S. leriifolia* as a physical barrier and by addressing low temperature as a physiological factor (i.e., ensuring that seedlings were cultivated in an environment with a warm, high temperature) were effective methods for the germination of this species. Hence, this study was designed to evaluate the effectiveness of CP incorporated with hydropriming on normal and uncovered seeds of *S. leriifolia* in overcoming the plant’s physical and physiological dormancy and improving its seed germination. The effect of these treatments was studied using SEM images, as well as biochemical and physiological experiments.

## 2 Material and methods

### 2.1 Seed preparation

In early June, *S. leriifolia* seeds were collected from Bajestan, located in Khorasan, Iran (34.31 N, 58.10 E). They were transferred to the Plant, Pests and Diseases Research Institute (PPDRI) in Tehran and incubated at 4°C.

### 2.2 Optical emission spectroscopy

The identification of reactive excited species was done in cleaner plasma from 200 to 950 nm at 80 and 100 W. Optical emission spectroscopy (OES) analysis was performed using Avantes Avaspec with a spectral resolution of 0.4 nm (Ara General Trading Ltc). Data were analyzed by Full XLS software based on the NIST reference website (Misra et al., 2015).

### 2.3 Hydropriming and cold plasma treatments

The seeds of *S. leriifolia* have hard coats, which consists the epicarp and mesocarp. The seeds were divided into four groups: normal seeds (i.e., dry and intact; NS), uncovered seeds (US) (the hard cover includes the epicarp; the mesocarp was removed, and the endocarp and testa of seeds remained on the seeds), hydro-primed normal seeds (HNS), and hydro-primed uncovered seeds (HUS). Based on the results of Sera et al. (2012), to prepare hydro-primed seeds of *S. leriifolia* with similar moisture levels, the normal and the uncovered seeds were incubated in sterilized water for 24 and 2 hours, respectively. At low pressures (i.e., 0.1 Pa), glow discharge, cold plasma ray (Plasma Cleaner, Satia Company, Iran) was operated at two power levels, 80 W and 100 W, and the sinusoidal waveform at 20 kHz for 120, 180, and 240 s. The seeds not treated with CP were utilized as the control. After CP treatment and hydropriming, the normal and the uncovered seeds were dried at 20–25°C for 24 and 2 hours, respectively, and were transferred to a growth chamber with a relative humidity of 75% and temperatures of 23 ± 1° C for 2 weeks. Following the germination of the seeds, they were checked daily, and properties were measured (Hussain et al., 2015).

### 2.4 Scanning electron microscopy

Five seeds of *S. leriifolia* from each treatment were used for external morphological analysis using SEM. Seeds were dehydrated through an ethanol series (50%, 70%, 85%, 95%, and 100%; each step 1 h). Seeds were then dried using a dryer. Seeds were mounted on stubs with the help of carbon sticky tape and sputter-coated with 125 nm of gold using a desktop Magnetron Sputtering device (Nanostructured Coatings Co. (NSC), Iran). Imaging, with 700 x magnification, was completed using a Hitachi SU-3500 microscope (Japan).

### 2.5 Germination percentage and germination rate

The recording of the number of germinated seeds was started after 3 days. Finally, the germination percentage (GP) was calculated based on the number of control germinated seedlings. The germination percentage was calculated using Equation 1:


(1) $$GP=\frac{n}{N}\times 100$$

where *n* is the number of germinated seeds on the *n*th day and *N* is the total number of seeds.

The germination rate (GR) was calculated based on the formula of Maguire (1962):


(2) $$GR=\frac{number\;of\;seeds\;germinated\;in\;3\;days}{total\;number\;of\;seeds}\;\times 100\%$$

### 2.6 Seed vigor I


(3) $$SVI=Shoot\;length\;\left(cm\right)+Root\;length\left(cm\right)\times Germination\frac{\left(\%\right)}{100}$$

### 2.7 Seed vigor II


(4) SVII=Seedling dry weight ×Germination (%)

### 2.8 Shoot length and dry weight measurement

The dry weight of the *S. leriifolia* seedlings was measured with a 0.001-g precision digital scale 14 days after the start of the experiment. The dry weight of these seedlings was recorded after oven drying (TARA TEB Company, model SANYO OMT oven) at 70°C for 48 h. The length of the seedlings was measured with a 1-mm precision ruler.

### 2.9 Biochemical measurements

All biochemical measurements were carried out when the radicles of *S. leriifolia* germinated seeds were visible to a length of 2 mm.

### 2.10 α-amylase activity assay

The method of Jones and Varner (1967) was used for the α-amylase activity assay. To prepare the enzyme extract of seedlings, 0.03 g of germinated seeds were ground in 3 ml of acetate buffer (pH 6) and incubated at 0–2°C for 5 h. The mixture was centrifuged at 5,000 g for 10 min. The reaction mixture contained 0.2 ml of the enzyme extract and 1 ml of fresh starch substrate [150 mg of insoluble starch + 600 mg of potassium dihydrogen phosphate (KH_2_PO_4_) + 200 µM of calcium chloride (CaCl_2_)], to which 100_ ml_ of water was added. The reaction mixture was then boiled for 1 min, cooled, and centrifuged for 10 min at 300 g, and incubated at 20°C for 5 min. Then the reaction was stopped using 1 ml of iodine reagent. [After preparation, we added potassium iodide (KI) + 600 mg of iodine (I_2_) + 100 ml of water. Then, to 1 ml of this solution, we added up to 100 ml of 0.05 N (Normal) hydrochloric acid (HCL)]. Finally, and after adding 5 ml water, α-amylase activity was measured at 620 nm using a Unico 2150 series advanced UV–Vis spectrophotometer.

### 2.11 Lipid peroxidation assay

For the estimation of lipid peroxidation, malondialdehyde (MDA) was measured according to the method of Heath and Packer (1968). Germinated seeds (0.2 g) were homogenized with 5 ml of trichloroacetic acid (TCA) with a concentration of 0.5% weight per volume (w/v). The mixture was heated at 95°C for 50 min and then quickly cooled in an ice bath. It was then centrifuged at 10,000 g for 10 min at 25°C. MDA concentration in the supernatant was determined by measuring absorbance at 600 nm and 532 nm.

### 2.12 Hydrogen peroxide assay

For measuring hydrogen peroxide (H_2_O_2_), 0.3 g of germinated seeds were homogenized in 2 ml of TCA 0.1% (w/v), 0.5 ml of 1 M KI and 0.25 ml of potassium phosphate buffer (10 mM, pH 7). After centrifugation at 12,000 g for 15 min at 4°C, the supernatant was incubated in the dark for 20 min. The concentration of H_2_O_2_ was measured at 390 nm using a spectrophotometer (Smart SpecTM Plus spectrophotometer, model hpd 011) based on the method of Junglee et al. (2014). The standard curve was used to calculate H_2_O_2_ content (Sigma-Aldrich).

### 2.13 Superoxide dismutase activity assays

The superoxide dismutase activity (SOD) was measured according the method of Misra and Fridovich (1972). The germinated seeds (200 mg) were homogenized in 5 ml of potassium phosphate buffer (100 mmol/l; pH 7.8) containing 0.1 mmol/l EDTA, 0.1% Triton X-100, and 2% polyvinyl pyrrolidone. The enzyme extract was filtered and centrifuged at 15,000 g for 15 min at 4 °C. Of the reaction mixture, 3 ml (total volume) contained 50 mmol/l sodium carbonate/bicarbonate buffer (pH 9.8), 0.1 mmol/l EDTA, 0.6 mmol/l epinephrine, and enzyme extract. Epinephrine was added at the end of the reaction, and adrenochrome formation in the following 4 min was recorded. Total SOD activity was measured as the amount of enzyme required to cause 50% inhibition of the rate of epinephrine oxidation at 475 nm, using a Unico 2150 series advanced UV-Vis spectrophotometer.

### 2.14 Catalase activity assay

A UV–Vis spectrophotometer (Unico 2150 series) was used to measure catalase activity in accordance with the procedure outlined by Beers and Sizer (1952) at a temperature of 25°C and a wavelength of 620 nm. The reaction mixture (5 ml) comprised 0.1 ml of enzyme extract and 2.9 ml of substrate solution (30% hydrogen peroxide in 50 mmol/l potassium phosphate buffer). The decomposition of H_2_O_2_ was stopped by adding 2 ml of potassium dichromate (5%). The enzyme activity was expressed as µmol H_2_O_2_ mg protein^–1^ min^–1^.

### 2.15 Total soluble protein assay

The total volume of soluble protein was measured using a Unico UV4 spectrophotometer. A standard curve was created using a series of concentrations of bovine serum albumin (BSA). Total soluble protein was measured using by combining 100 μl of extract and 3 ml of Bradford’s solution, and samples were vortexed to ensure proper mixing. After 20 minutes, each sample’s protein concentration was measured using an advanced UV–Vis spectrophotometer from the Unico 2150 series at 595 nm.

### 2.16 Statistical analysis

The experiment of this study was conducted as a completely randomized design in a factorial arrangement with 24 treatments, three replications with 150 seeds in each replication. Six Petri dishes, each containing 25 seeds, were used in each replication. The data obtained were subjected to analysis of variance (ANOVA) using SAS software version 9.1 (SAS Institute, Cary, NC, USA). The significant differences among the treatments were determined by least significant difference (LSD) multiple range tests (*p*< 0.05).

## 3 Result and discussion

### 3.1 Optical emission spectroscopy of plasma

**Figure 1:**
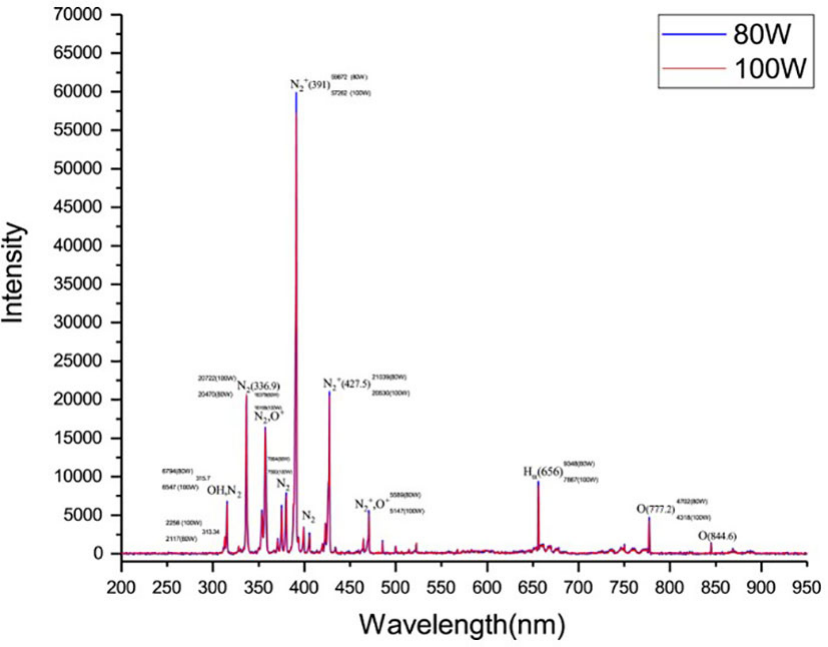
The optical emission spectrum of low-pressure glow discharge in the air at the power of 80 (left) and 100 (Right) W.

Cold plasma exposed to low-pressure air in the plasma cleaner showed that different types of reactive excited species, such as dinitrogen (N_2_^+^), nitrogen (N_2_), hydroxide (OH), oxygen (O_2_), oxonium ions (O^+^) and hydrogen alpha (Hα) were produced, and the most reactive excited species was nitrous oxide (NOS) (**Figure 1**) . The study of emission spectra from 200 to 950 nm at 80 and 100 W resulted in an observation of the most intensive peak with the creation of N_2_^+^ at 391 nm for both, but the intensity of this active species at 80 W (59872) was higher than 100 (57262) W (**Figure 1**). Similarly, reactive nitrogen species (RNS), e.g., nitrogen dioxide (NO_2_), nitric oxide (NO), nitrate (NO^–^_3_), nitrite (NO^–^_2_), and peroxynitrite (PON), reactive oxygen species (ROS), e.g., O_2_^–^, O_2_, ozone (O_3_), H_2_O_2_, and OH and ultraviolet (UV) photons were reported using CP in the air (Selwyn et al., 2001; Laroussi and Leipold, 2004; Song et al., 2020). 

### 3.2 Seed surface properties by SEM

**Figure 2:**
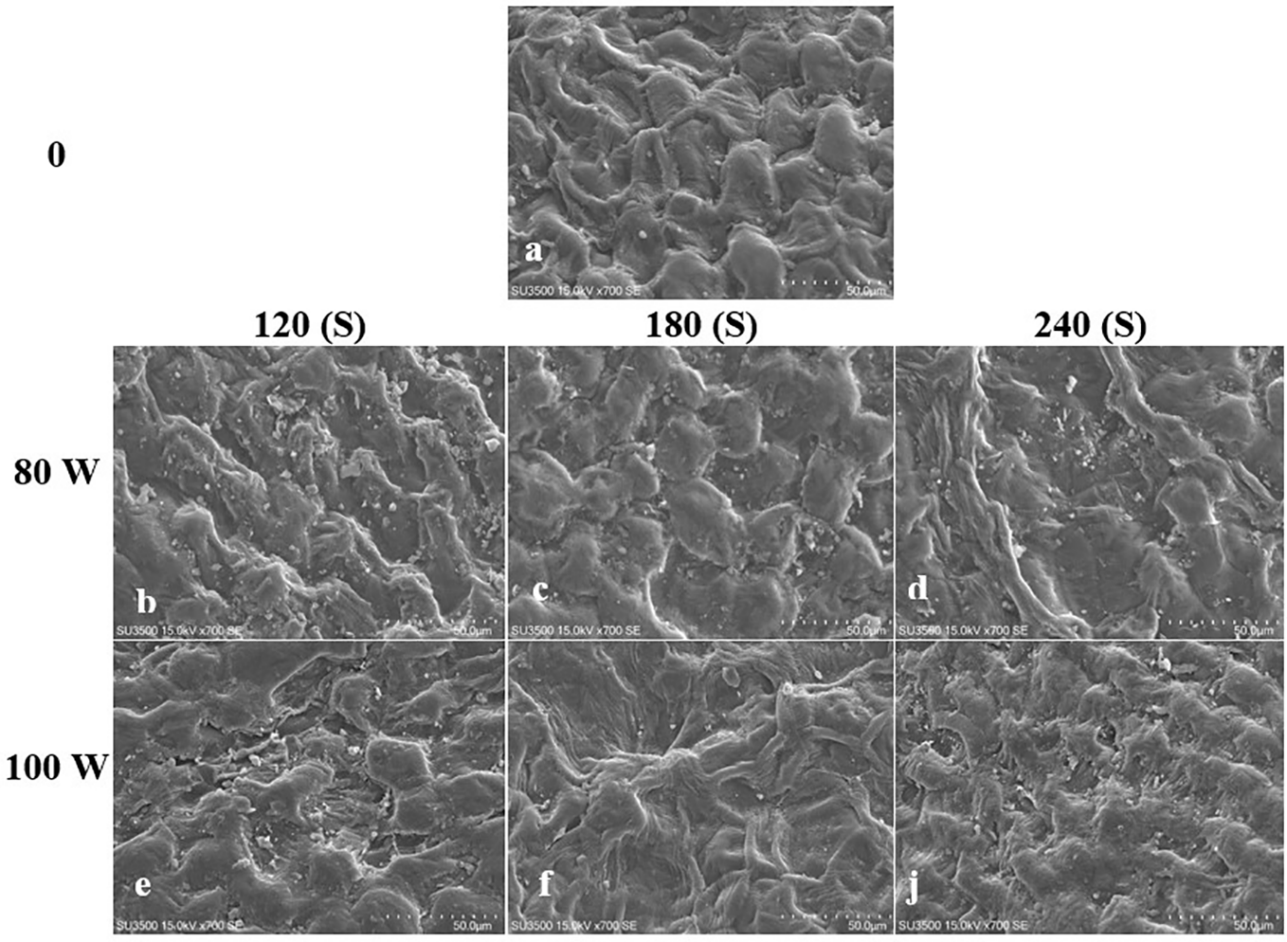
Scanning Electron Microscope (SEM) images of the normal seed (NS) surface of *S. leriifolia* under cold plasma. **(A)** NS, **(B)** NS in 80 W 120s, **(C)** NS in 100 W 120s, **(D)** NS in 80 W 180s, **(E)** NS 100 W 180s, **(F)** NS in 80 W 240s, **(J)** NS 100 W 240s.

**Figure 3:**
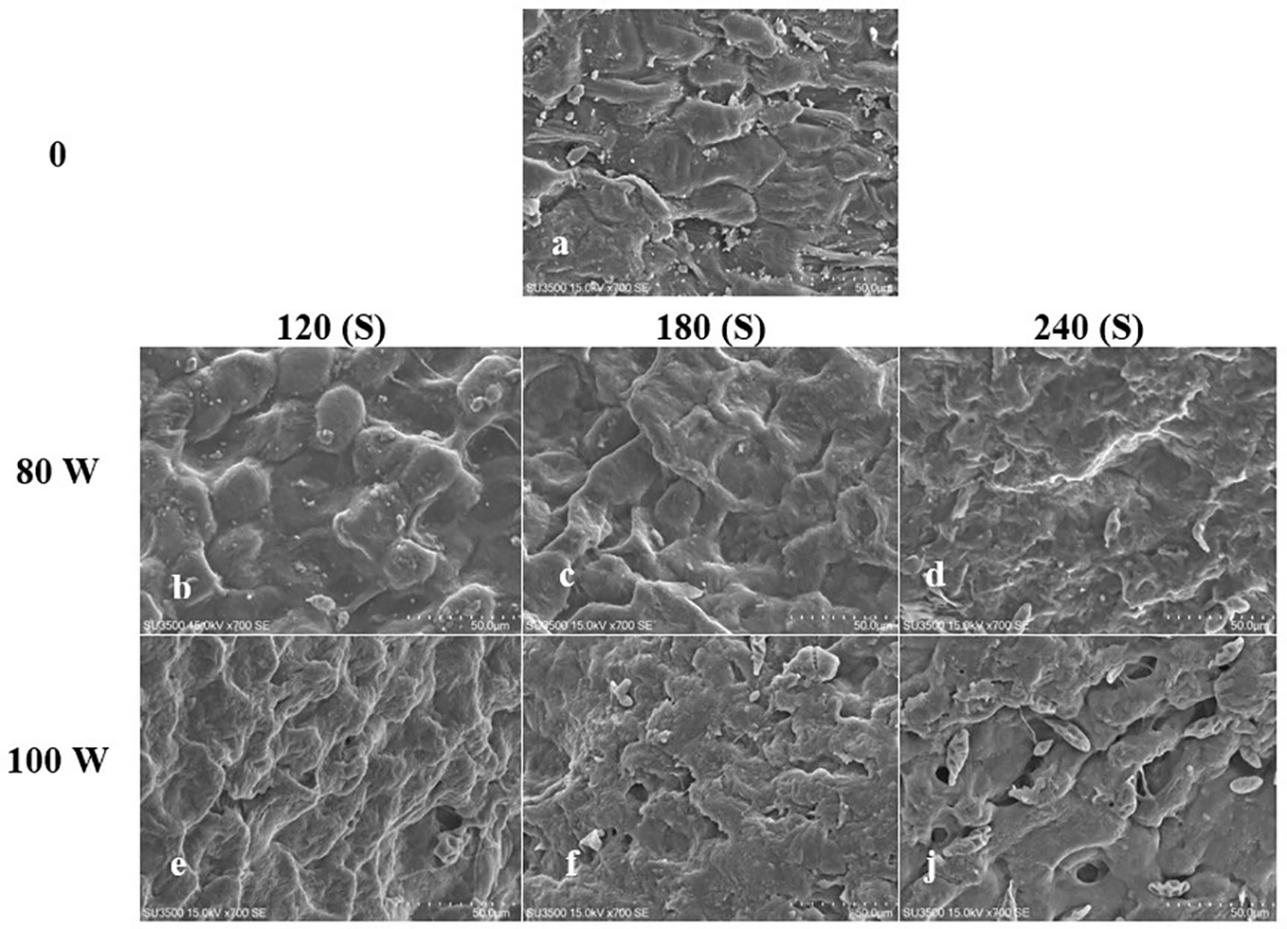
Scanning Electron Microscope (SEM) images of the hydro-primed normal seeds ONS) surface of *S. leriifolia* under cold plasma. **(A)** HNS, **(B)** HNS in 80 W 120s, **(C)** HNS in 100 W 120s, **(D)** HNS in 80 W 180* **(E)** HNS in 100 W 180s, **(F)** HNS in 80 W 240s, **(J)** in 100 W 240s.

**Figure 4:**
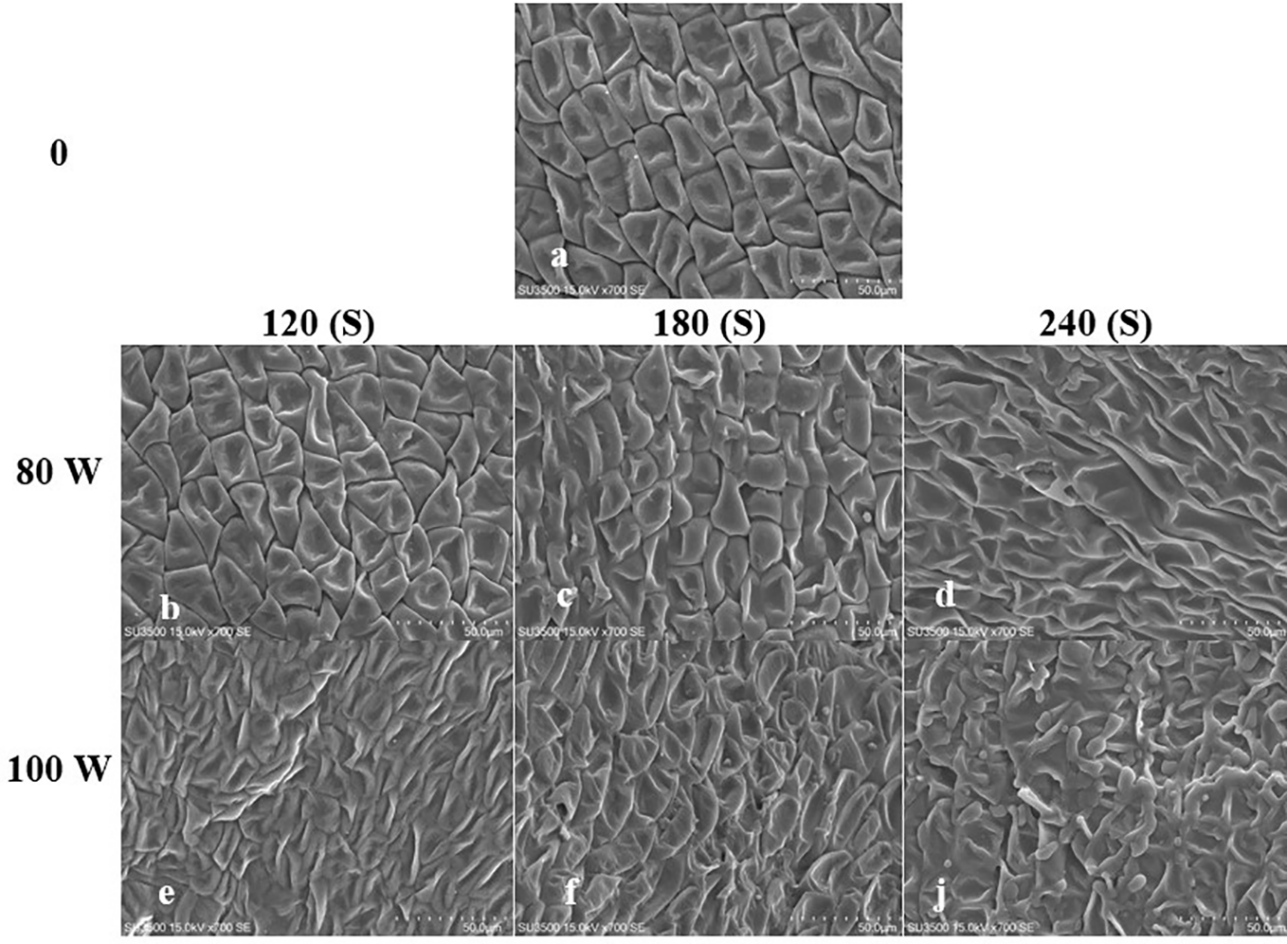
Scanning Electron Microscope (SEM) images of the uncovered seed (US) surface of S. leriifolia under cold plasma. **(A)** uncovered seeds (US), **(B)** US in 80 W 120s, **(C)** US in 80 W 180s, **(D)** US in 80 w 240s, **(E)** US in 100 W 120s, **(F)** US in 100 W 180s, **(J)** US in 100 W 240s.

**Figure 5:**
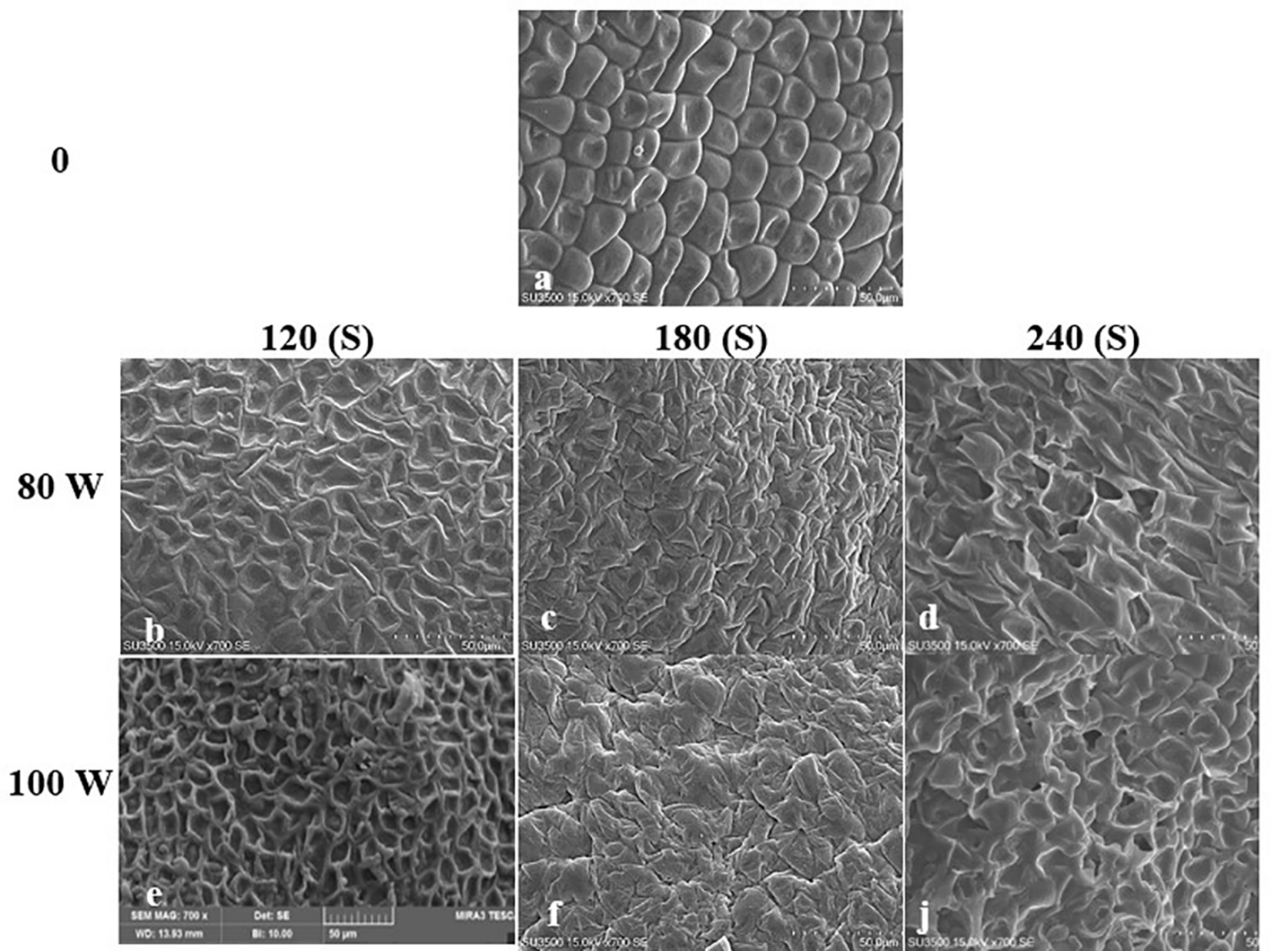
Scanning Electron Microscope (SEM) images of the hydro-primed uncovered seed (HUS) surface of *S leriifolia* under cold plasma. **(A)** Hydro-primed uncovered seeds (HUS), **(B)** HUS in 80 W 120s, **(C)** HUS in 80 W 180s, **(D)** HUS in 80 W 240s, **(E)** HUS in 100 W 120s, **(F)** HUS in W 180s, **(J)** HUS in 100 W 240s.

The effect of RF-cold plasma, incorporating hydropriming, on changes in *S. leriifolia* seed morphology, as detected using SEM images, is shown in **Figures 2**–**5**. The significant etching effect in seed surfaces of NS, US, HNS, and HUS in terms of CP exposure can be observed clearly (**Figures 2**–**5**). The regular mesh-like structure was identified in NS, US, HNS, and HUS- free-cold plasma. These structures were significantly destroyed by increasing RF-cold plasma power and the exposure time, so the seed surface morphology was more changed, and the tissue became more permeable. It seems that the incidence of destroyed mesh-like structures in the hydro-primed seeds, i.e., HNS and HUS, was higher than that in the dry seeds (**Figures 2**–**5**). In these experiments, like other studies, the incidence of more severe changes in the seed surface morphology increased as the CP treatment time increased (**Figures 2**–**5**) (Li et al., 2017). Therefore, the degree of seed coat deformation and the size of the cracks observed are proportional to the plasma exposure time (Le et al., 2022). As the surface of normal and uncovered seeds was different altogether, the changes in the seed surface morphology were also different. The effect of CP, as a destroyer of conspicuous mesh-like structures surface in some SEM images of NS, US, HNS, and HUS, was uneven (**Figures 2**–**5**). Similarly, the inconsistent etching of seed surfaces of barley and pea seeds was reported (Stola´rik et al., 2015; Park et al., 2018). Park et al. (2018) suggested that this can be related to the chemical reaction of reactive species of CP with the seed surface and the differences in the energy distribution of CP.

### 3.3 Germination percentage and germination rate

The effects of different treatments on the seed germination and growth traits of *S. leriifolia* seedlings are presented in **Table 1**. The statistical analysis of the germination percentage (GP) and germination rate (GR) of *S. leriifolia* seeds showed that the individual effects of SS, CP power and time, the two–way interactions between SS × CP time, and power of CP × time of cold plasma had a significant influence on them. However, the two-way interactions between SS and power of CP and the three-way interactions showed a non-significant effect on GP and GR (**Table 1**). Based on the results, removing the hard coat (uncovered seeds) and hydropriming had a 1.3-times impact on GP and GR compared with the NS. Removing the seed coat increases seed germination as it allows for increased water uptake by seeds (which in turn changes their physical dormancy) because the seed coat regulates dormancy partly by restricting the diffusion of O_2_ into the embryo of seeds (Stabell et al., 1998; Tezuka et al., 2012). The seed coat of *S. leriifolia* inhibits water and O_2_ uptake and the activity of enzymes, which are effective during seed germination. The GP of uncovered seeds (US, and HUS) was increased more than in the NS and HNS seeds with increasing time of exposure to CP (**Table 2**). Similarly, the GR in the uncovered seeds of *S. leriifolia* was higher than in the normal seeds (NS and HNS) (**Table 2**). The GP and GR of all seeds were increased by increasing the power and exposure time of CP (**Tables 2**, **3**). The results indicated that prolonged exposure time of HU seeds to CP for 240 s increased GP and GR significantly, by 202.9% and 205.2%, respectively, compared with the control seeds (NS) (**Table 2**). The lowest GP and GR were observed for NS under 120s CP treatment (**Table 2**). The main mechanisms for effective seed germination include increased wetting and water uptake by the seed *via* improving the hydropholicity of the seed coat surface and so increasing its water uptake. The results of the SEM analysis showed that the untreated (control) seed surface underwent morphological changes after being exposed to 100 W of CP for 240s. The surface of the treated seeds seemed smoother, and even minute holes were observed (**Figures 2**–**5**). Our findings also agree with other reports in which CP is shown to increase the GP and GR of treated seeds (Nishime et al., 2020; Le et al., 2022).

**Table 1:** Variance analysis of the effect of seed situation, cold plasma (CP) power and exposure time of cold plasma on germination percentage and rate, seedling length, and SVI and SVII of *S. leriifolia* seeds.

S.O.V	df	MS					
		Germination (%)	Germination rate	Seedling length	Seedling dry weight	SVI	SVII
SS	3	24.85**	25.32**	5.40 **	13.31**	13.99**	26.11**
CP power	1	29.43**	28.35 **	0.05ns	0.01ns	1.36ns	1.59ns
CP time	3	30.22**	29.62**	12.40**	7.06**	9.68**	36.34**
SS × CP power	3	0.41 ns	0.24 ns	0.48ns	2.61ns	3.76*	11.69**
SS × CP time	9	6.92 **	7.11 **	1.99 ns	4.27**	4.71**	7.21 **
CP time × CP power	3	4.30**	4.04**	0.25ns	3.53*	2.13ns	0.85ns
SS × CP time × CP power	9	0.21ns	0.19ns	1.73ns	2.78**	2.43*	3.94**
Error	64	1578.61	90.1	4.93	0.001	1.08	9637
CV %		18.8	18.85	13.16	22.87	28.94	21.23

* and ** significant at 1% and 5% respectively and ^ns^ non-significant different. CP, cold plasma; df, degrees of freedom; SS, seed situation; SV, seedling vigor; SOV, Source Of Variation; MS, Mean Squares; CV, Coefficient of Variation.

**Table 2:** The effect of seed situation and cold plasma exposure time on the germination percentage and rate and superoxide dismutase (SOD) activity of *S. leriifolia* seeds.

Seed situation	Cold plasma exposure time (seconds)			
	0	120	180	240
	Germination percentage (%)			
NS	24.0±(0) d-f	14.0±(2.19) h	22.0±(4.2) e-g	22.00±(8.29) eg
US	32.0±(0) bc	25.3±(4.13) df	20.0± (5.06) fg	33.3±(13.78) b
HNS	29.3±(5.47) bd	24.7±(5.89) df	18.0±(5.51) gh	26.7±(4.13) ce
HUS	32.0±(0) bc	24.7±(3.93) df	24.7±(3.93) df	48.7±(8.55) a
	Germination rate (seed per day)			
NS	5.7±(0) dg	3.3±(0.52) h	5.2±(1.0) eg	5.2±(1.98) eg
US	7.6±(0) bc	6.0±(0.98) df	4.9±(1.11) fg	7.9±(3.28) bc
HNS	7.0±(1.3) bd	5.9±(1.4) df	4.4±(1.3) gh	6.3±(0.98) df
HUS	7.6±(0) bc	5.9±(0.93) df	5.9±(0.93) df	11.7±(1.97) a
	SOD activity (U/mg)			
NS	0.230±(0.009) ef	0.236±(0.003) ce	0.231±(0.003) df	0.243±(0.006) ab
US	0.229±(0.001) ef	0.243±(0.005) ab	0.234±(0.003) cf	0.238±(0.008) bc
HNS	0.228±(0.011) f	0.246±(0.006) a	0.239 ±(0.01) bc	0.234±(0.004) cf
HUS	0.228±(0.010) f	0.238±(0.005) bc	0.244±(0.012) ab	0.238 ±(0.007)bc

Values represent mean ± S.E. Different letters indicate significant differences at the 1% level according to the LSD test. NS, normal seeds; US, uncovered seeds; HNS, hydro-primed normal seeds; HUS, hydro-primed uncovered seeds; SOD, superoxide dismutase.

**Table 3:** The effect of power and exposure time of cold plasma on the germination percentage and rate and superoxide dismutase (SOD) activity of *S. leriifolia* seeds.

Cold plasma power (W)	Cold plasma time (seconds)			
	0	120	180	240
	Germination percentage (%)			
80	29.3±(4.29) b	19.7±(5.52) d	17.7±(3.17) d	27.7±(10.1) bc
100	29.3±(4.29) b	24.7±(6.11) c	24.7±(4.12) c	37.7±(15.01) a
	Germination rate			
80	7.0±(1.02) b	4.7±(1.31) d	4.3±(0.076) d	6.7±(2.54) bc
100	7.0±(1.02) b	5.9±(1.46) c	5.9±(0.033) c	9.0±(2.57) a
	SOD activity (U/mg)			
80	0.228±(0.07) e	0.244±(0.005) a	0.242±(0.10) ab	0.243±(0.005) a
100	0.230±(0.009) de	0.238±(0.006) bc	0.232±(0.002) cd	0.232±(0.004) cd

Values represent mean ± S.E. Different letters indicate significant differences at the 5% level according to the LSD test. LSD, least significant difference; SOD, superoxide dismutase.

These modifications can have a positive effect on improving the water permeability and wettability of *S. leriifolia*. These findings correspond with the results of Nishime et al. (2020) in winter wheat seeds treated by argon and helium dielectric barrier discharge (DBD), Tong et al. (2014) in *Andrographis paniculata* treated with air plasma, and Stola´rik et al. (2015) in peas exposed to low-temperature plasma.

Seed germination and related processes depend on the water potential of a substrate (Pereira et al., 2012). The seed coat acts as a structural barrier against the entry of any external factors, and regulates the gas and water exchange required for seed germination (Finch-Savage and Leubner-Metzger, 2006; Baskin and Baskin, 2014). Therefore, any modification to the seed coat might enhance the hydrophilic properties of the seed and, ultimately, enhance water intake, which indirectly improves seed germination (Bormashenko et al., 2012; Le et al., 2022). The observed increased germination and other traits of *Salvia* seeds treated with high power (100 W) for longer exposure times (240 s) in HUS related to the improvement in water uptake and wettability of seeds, which was consistent with the results of Nishime et al.’s study (Nishime et al., 2020) on wheat and Stola´rik et al.’s study (Stola´rik et al., 2015) on peas. Besides, the penetration of reactive species into the porous seed coat leads to a reaction with the inner cellular components and causes metabolic changes in the seeds (Sera et al., 2010) that have been shown to have a positive effect on cell function. Our observation also agrees with a recent report, which indicated that a CP exposure time of 240 s promoted seed germination and seedling growth in mung bean. Their findings showed that the high levels of NO in the excited species of CP induced up-regulation of GA_3_ and improved germination (Le et al., 2022).

Correlation analysis (**Table 4**) reflected significant positive Pearson correlations between GP and GS, seedling length, seedling dry weight, SVI, and SVII in normal and uncovered *S. leriifolia* seeds under CP and hydropriming, whereas the correlations between GP and H_2_O_2_, SOD, and CAT were negative. These results indicated that by improving GP and GR by removing the seed cover, CP and hydropriming has a positive effect on growth factors, and the positive effect of CP, that is modification of the seed surface so that there is increased water uptake, are depend on the power and exposure time of CP, as well as the type of seed surface (Srisonphan, 2018).

**Table 4:** Pearson correlation matrix for morphological, biochemical, and antioxidant enzyme activity in normal and uncovered *S. leriifolia* seeds under cold plasma treatment (80 and 100 W with an exposure time of 0, 120, 180, and 240 s) and hydropriming.

	Germination percentage	Germination rate	Seedling length	Seedling dry weight	SVI	SVII	MDA	H2O2	SOD
**Germination rate**	0.99^**^	1							
**Seedling length**	0.11^ns^	0.1^ns^	1						
**Seedling dry weight**	0.44^**^	0.44^**^	0.29^*^	1					
**SVI**	0.47^**^	0.49^**^	0.24^*^	0.35^**^	1				
**SVII**	0.58^**^	0.57^**^	0.35^**^	-0.33^**^	0.64^**^	1			
**MDA**	0.27^*^	0.27^**^	-0.3^**^	0.02^ns^	-0.16^ns^	-0.11^ns^	1		
**H_2_O_2_**	-0.24^*^	0.24^*^	-0.33^**^	0.02^ns^	-0.2^ns^	-0.16^ns^	0.87^**^	1	
**SOD**	-0.21^*^	-0.22^*^	-0.23^**^	-0.23^*^	-0.16^ns^	-0.21^*^	-0.02^ns^	-0.01^ns^	1
**CAT**	-0.23^**^	-0.23^**^	-0.39^**^	-0.15^ns^	-0.18^ns^	-0.39^**^	0.12^*^	0.21^*^	0.62^**^

**and * indicate significantly of correlation at the 1 and 5%, respectively.

CAT, catalase; H_2_O_2_, hydrogen peroxide; MDA, malondialdehyde; SOD, superoxide dismutase; SVI, seed vigor; SVII, seed vigor II.

### 3.4 Seedling length and dry weight

**Figure 6:**
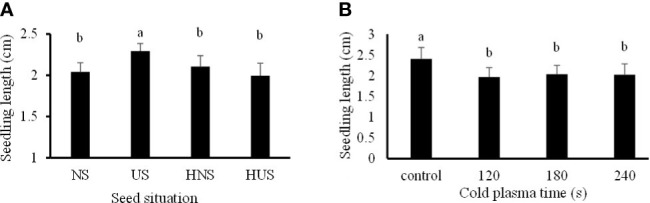
The effect of seed situation **(A)** and cold plasma exposure time **(B)** on seedling length of *S. leriifolia* genninated seeds. Normal seeds (NS), Uncovered seeds (US), Hydro-primed normal seeds (HNS) and Hydro-primed uncovered seeds (HUS). Values represent mean ± S.E. Different letters indicate significant differences at the 1% level according to the LSD test.

The statistical analysis (**Table 1**) showed that just the individual effect of SS and CP time significantly affect seedling length. The seedling length of the uncovered seeds (US) was 2.29 cm, significantly increased by 11.02%, 8.2%, and 13.07%, compared with the NS (control) seeds, HNS, and HUS, respectively (**Figure 6A**). Meanwhile, there were no significant differences among HNS, HUS, and NS (control) in the seedling length of *S. leriifolia* plants (**Figure 6A**). Our results showed that seed hydropriming had no significant effect on the seedling length of *S. leriifolia* plants (**Figure 6A**). The seedling length decreased by 18.26%, 15.22%, and 15.8% in CP exposure times of 120, 180, and 240 s compared to the control, respectively (**Figure 6B**). Similarly, Kordas et al. (2015) reported that air CP (8 kV for 10 s) had no significant effect on the length and dry weight of wheat. However, an increase in these traits was observed in tomato (Magureanu et al., 2018) and *Carthamus tinctorius* L. (Dhayal et al., 2006).

The statistical analysis showed that the dry seedling weight of *S. leriifolia* plants was significantly affected by the individual effect of SS, CP time, and the two-way interactions between SS and CP time (**Table 1**). Moreover, it was significantly affected by two-way interactions between CP power and CP time, and three-way interactions between them (**Table 1**). The highest seedling dry weight was measured as 0.0333 mg using 100 W of CP power for 240 s in HUS. Therefore, it increased by 221% compared with the same treatment (HUS) in the control (**Table 5**). Similar results have been reported, showing an increase in seedling height and dry weight in soybeans and wheat after the use of CP (Ling et al., 2014; Zahoranová et al., 2016). Previous studies confirmed that the effect of plasma on plant growth is a result of changes in the amount of growth hormones (through regulating auxin transport), and other physiological processes [such as the cell division process in a meristem region and cellular differentiation (Iranbakhsh et al., 2018; Le et al., 2022)]; increased nitrogen nutrient levels (by producing reactive nitrogen species, such as NO_2_ and NO_3_) (Sivachandiran and Khacef, 2017; Le et al., 2022), and the activation of growth-related gene expression (Adhikari et al., 2020); and improvement of mobilized seed reserves, and seed reserve depletion percentage (because dry seedling weight is significantly related to these parameters) (Soltania et al., 2006; Dobrynin et al., 2009).

**Table 5:** The effect of power and exposure time of cold plasma and seed situation on dry weight, SVI, and SVII of *S. leriifolia* germinated seeds.

Experimental treatments			Seedling dry weight (g per plant)	SVI	SVII
CP power (W)	CP time (s)	Seed situation			
0 (control)		NS	0.0133 ± (0.002) f-i	0.36 ± (0.02) ej	58.3 ± (13.5) dg
		HNS	0.017 ± (0.001) c-i	0.25 ± (0.07) ij	33.9 ± (11.9) ik
		US	0.012 ± (0.001) h-i	0.31 ±(0.05) gi	47.8 ± (3.6) fg
		HUS	0.0107 ± (0.001) i	0.34 ± (0.06) fj	55.7 ± (9.1) dh
80 W	120	NS	0.0247 ± (0.003) b	0.66 ± (0.13) b	86.6 ± (7.7) bc
		HNS	0.0163 ± (0.002) di	0.62 ± (0.14) bc	72.7 ± (10.2) bd
		US	0.0223 ± (0.009) bd	0.59 ± (0.03) bd	60.4 ± (28.0) dg
		HUS	0.0183 ± (0.006) bh	0.48 ± (0.19) bh	63.1 ± (30.1) df
	180	NS	0.0163 ± (0.002) di	0.57 ± (0.15) be	72.7 ± (18.1) bd
		HNS	0.015 ± (0.005) ei	0.4 ± (0.12) dh	36.6 ± (3.0) hk
		US	0.0167 ± (0.003) ci	0.26 ± (0.06) ij	28.6 ± (0.7) jk
		HUS	0.0193 ± (0.009) bf	0.32 ± (0.03) fi	38.2 ± (18.1) hk
	240	NS	0.0223 ± (0.009) bd	0.64 ± (0.12) bc	92.7 ± (4.5) b
		HNS	0.013 ± (0.001) fi	0.48 ± (0.25) bh	56.1 ± (4.0) df
		US	0.023 ± (0.005) bc	0.53 ± (0.19) bf	25.3 ± (2.3) k
		HUS	0.0167 ± (0.001) ci	0.66 ± (0.5) b	70.8 ± (1.0) cf
100 W	120	NS	0.0247 ± (0.002) b	0.64 ± (0.64) bc	86.6 ± (7.7) bc
		HNS	0.014 ± (0.001) ei	0.44 ± (0.06) ci	63.3 ± (0.6) df
		US	0.023 ± (0.003) bc	0.29 ± (0.09) gi	49.5 ± (14.8) ei
		HUS	0.0167 ± (0.001) ci	0.28 ± (0.18) hj	24.3 ±(2.1) k
	180	NS	0.0163 ± (0.003) di	0.57 ± (0.15) be	72.7 ± (18.1) bf
		HNS	0.0143 ± (0.001) ei	0.22 ± (0.11) ij	45.5 ± (7.5) fj
		US	0.0133 ± (0.003) fi	0.47 ± (0.23) bh	43 ± (19.3) gk
		HUS	0.018 ± (0.000) ch	0.5 ± (0.37) bg	53 ± (21.1) dh
	240	NS	0.0223 ± (0.029) bd	0.64 ± (0.12) bc	92.7 ± (4.5) b
		HNS	0.012 ± (0.000) hi	0.37 ± (0.01) ej	56 ± (8.0) dh
		US	0.0203 ± (0.006) be	0.25 ± (0.03) ij	68.9 ± (8.8) cf
		HUS	0.0333 ± (0.006) a	0.89 ± (0.01) a	123.2 ± (0.5) a

Values represent mean ± S.E. Different letters indicate significant differences at the 5% level according to the LSD test. CP, cold plasma; NS, normal seeds; US, uncovered seeds; HNS, hydro-primed normal seeds; HUS, hydro-primed uncovered seeds; LSD, least significant difference.

### 3.5 Seed vigor I and seed vigor II

The statistical analysis (**Table 1**) showed that SV I and II of the *S. leriifolia* plant were significantly affected by the individual effect of SS and CP time, the two-way interactions between SS and CP power, and SS and CP time, and the three–way interactions of them (**Table 1**). However, although initially vigor index I and II of different CP power and exposure times was observed in NS, but the highest was found when the hydro-primed uncovered seeds (HUS) were treated with 100 W of CP for 240 s (**Table 5**). This percentage increase was 221% and 261% for SVI and SVII, compared with the control, respectively (**Table 5**). The results showed that removing the cover and hydropriming had no effect on SVI and SVII, but that CP with both powers could influence the germination and growth parameters in most of the treatment combinations (**Table 5**). The results indicate that CP mostly affected the physiological dormancy of *S. leriifolia* seeds and that changes in SVI and SVII depend on the plasma treatment conditions. Correlation analysis (**Table 4**) reflected significant positive Pearson correlations between SVI and SVII, GP and GS, seedling length, and seedling dry weight (**Table 4**). These indexes depend on the seedling length and dry weight. Therefore, any change in these parameters can also affect SVI and SVII. It seems that the increase in SVI and SVII relates to the increase in length and dry weight of *S. leriifolia* seedlings in their corresponding treatments (Ling et al., 2014; Tong et al., 2014; Stola´rik et al., 2015). The negative correlations between SVII and the activity of SOD and catalase (CAT) indicate that with the improvement of germination conditions because of CP use, the activity of these enzymes decreases (**Table 4**). Despite the increase in MDA and H_2_O_2_ in the treated seeds, the correlation analysis showed that this increase had no effect on SVI, SVII, and the germination process.

### 3.6 α-amylase activity

The statistical analysis (**Table 6**) revealed that the individual effect of SS and CP power and time significantly affects α-amylase activity. The two-way interactions between the power of CP and time of CP had a highly significant (*p*< 0.001) effect on the α-amylase activity (**Table 6**).

**Table 6:** Variance analysis of the effect of seed situation, cold plasma (CP) power, and exposure time of cold plasma on α-amylase, MDA, H_2_O_2_, and antioxidant enzyme activities of *S. leriifolia* seeds.

SOV	df	MS				
		α-amylase	MDA	H_2_O_2_	SOD	CAT
Seed situation (SS)	3	4.96^**^	99.24^**^	2.85^*^	0.76 ^**^	4.39^**^
CP power	1	4.57 ^*^	8216.45^**^	112.28^**^	30.04^**^	33.65^**^
CP time	3	19.36^**^	4361.82^**^	69.87^**^	21.76^**^	70.11^**^
SS × CP power	3	1.94^ns^	74.53^*^	7.99^**^	2.05^ns^	17.39^**^
SS × CP time	9	1.37 ^ns^	9.07 ^**^	1.46 ^ns^	4.43^**^	9.17^**^
CP time × CP power	3	7.05^**^	1980.68^**^	21.01^**^	7.01^**^	5.19^**^
SS × CP time × CP power	9	1.63^ns^	86.53^**^	2.19^*^	1.09^ns^	4.49^**^
Error	64	0.051	0.0002	0.0018	0.0021	0.0008
CV%		11.34	1.15	2.47	2.42	8.43

*and** significant at 1% and 5%, respectively and ^ns^ non-significant different. CP, cold plasma; df, degrees of freedom; SOD, superoxide dismutase; CAT, catalase; MDA, malondialdehyde; SOV, Source Of Variation.

**Figure 7:**
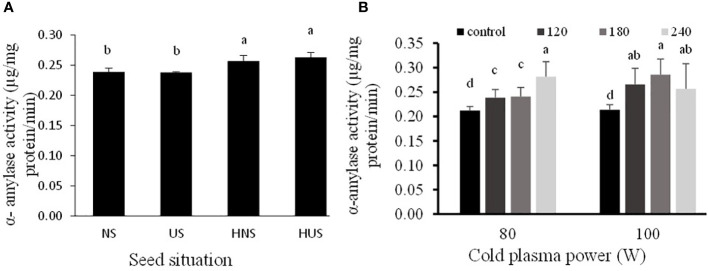
The individual effect of seed situation **(A)** and power of cold plasma **(B)** on α-amylase activity of *S. leriifolia* germinated seeds. Values represent mean ± S.E. Different letters indicate significant differences at the I and 5% level for seed situation and power of cold plasma according to the LSD test, respectively.

The activity of α-amylase enzyme in the hydro-primed *S. leriifolia* germinated seeds, including HNS, and HUS was higher than in the dry seeds (NS and US) (**Figure 7A**). The activity of α-amylase enzyme in the germinated *S. leriifolia* seeds increased significantly with the increase of exposure time of 80 and 100 W CP as compared with the control (**Figure 7B**). However, although 100 W of CP increased enzyme activity after 240 s, α-amylase enzyme activity after 240 s was lower than before the 180 s time point. So, the highest activity of this enzyme in comparison with the control (24.82 µg/mg protein) was observed in the 80 and 100 W CP treatment for 240 and 180 s as 0.282 and 0.288 µg/mg protein, respectively (**Figure 7B**). The internal α-(1–4) glycosidic linkages in starch are hydrolyzed by α-amylases that are released by the cells of the seed’s aleurone layer. Complex starches break down into compounds with low molecular weights, such as glucose, maltose, and maltotriose units (Rajagopalan and Krishnan, 2008; Sun et al., 2015). Therefore, this system offers metabolites and energy for seed germination and seedling early development. The uncovering of *S. leriifolia* seeds did not affect the activity of the α-amylase enzyme. In contrast, hydropriming both normal and uncovered seeds for 24 h and 2 h increased this enzyme activity (**Figure 7** **A**). The positive effect of hydropriming on α-amylase activity and acceleration of starch degradation was reported in rice (Illangakoon et al., 2016). Enhancing the activity of amylase enzyme by increasing the power and exposure time of CP by 1.5 times, with 80 W for 240 s and 100 W for 180 s, compared with the control, related to the changes in the activation of hormones and particles produced because of CP treatment. The embryo of seeds synthesizes gibberellin (GA) hormones, which the synthesis and secretion of α-amylase are activated by (Sun, 2008; Mildaziene et al., 2019). The results obtained by Finkelstein (2010) show that CP treatment can induce the activation of gibberellin before amylase activity begins. Furthermore, increasing the activity of amylase in *S. leriifolia* seeds can be attributed to the chemical reaction of reactive species produced with CP, more precisely nitric oxide (NO) (Miransari and Smith, 2014; Gómez-Ramírez et al., 2017).

### 3.7 Malondialdehyde and hydrogen peroxide contents

**Figure 8:**
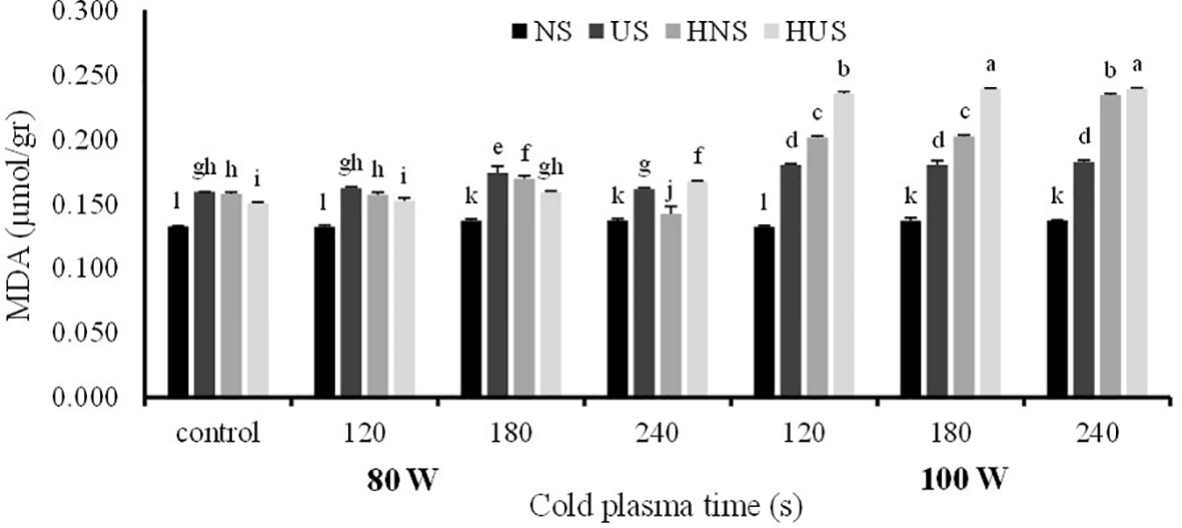
The effect of power and exposure time of cold plasma and seed situation on MDA content of *S. leriifolia* germinated seeds. Normal seeds (NS), Uncovered seeds (US), Hydro-primed normal seeds (HNS) and Hydro-primed uncovered seeds (HUS). Values represent mean ± S.E. Different letters indicate significant differences at the 1% level according to the LSD test.

**Figure 9:**
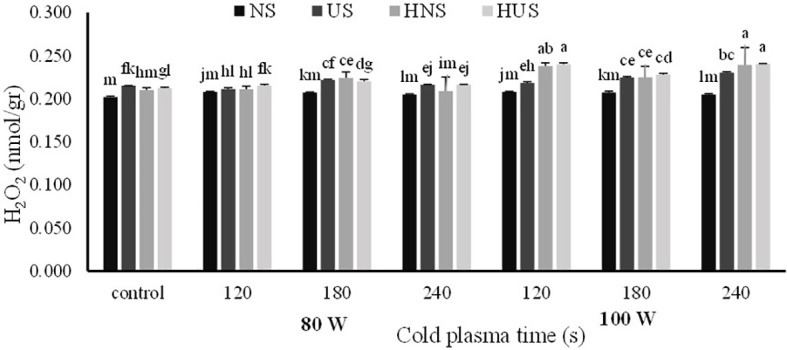
The effect of power and exposure time of cold plasma and seed situation on H_2_0_2_ content of *S. leriifolia* germinated seeds. Normal seeds (NS), Uncovered seeds (US), Hydro-primed normal seeds (HNS) and Hydro-primed uncovered seeds (HUS). Values represent mean ± S.E. Different letters indicate significant differences at the 5% level according to the LSD test.

The statistical analysis of malondialdehyde (MDA) and H_2_O_2_ contents of *S. leriifolia* germinated seeds showed (**Table 6**) that the individual effect of SS, CP power and time, and two- and three-way interactions among them had a significant effect (*p*< 0.001) on their contents, but two-way interactions between SS and exposure time of CP showed a non-significant effect on H_2_O_2_ content. The results indicated that MDA content increased in US and HNS by increasing the power and exposure time of CP (**Figure 8**). However, the changes in MDA content by increasing the exposure time of CP at 80 W in HUS were slower, but the maximum MDA content was obtained in these seeds at all exposure times of CP (i.e., 120 s to 240 s) (**Figure 8**). Instead, the minimum content of MDA with lower levels of changes was obtained in NS by increasing the power and exposure time of CP (**Figure 8**). The results of three-way interactions between SS, power of CP and exposure time of CP on the H_2_O_2_ content showed that similarly to MDA content, the most minor content of H_2_O_2_ with lower changes was recorded in NS by increasing the power and exposure time of CP (**Figure 9**). The results showed that H_2_O_2_ content in the US, HNS, and HUS by increasing the power and exposure time of CP increased, so the highest was recorded in HNS and HUS for 120 s and HUS for 240 s (**Figure 9**).

H_2_O_2_ and MDA are considered to be oxidative stress indicators in plants under (biotic and abiotic) stress conditions (Zulfiqar et al., 2021; Li et al., 2021). Similarly, other researchers observed an increase in MDA content in CP treatments (Jiang et al., 2014; Rahman et al., 2018; Los et al., 2019; Cui et al., 2019; Adhikari et al., 2020). Increased MDA accumulation was observed by enhancing the power and exposure time of CP in germinated seeds of *S. leriifolia* plants. Moreover, its accumulation was higher in uncovered and hydro-primed seeds. Similarly, changes in MDA content were reported in the CP-treated wheat and rapeseed seeds (Ling et al., 2015; Guo et al., 2017; Li et al., 2017). In other words, the lower MDA content at 80 W of CP indicated that the cell membrane stability at this power was better than at 100 W of CP in the same conditions because MDA is the end product of the peroxidation of unsaturated fatty acids in cell membranes. So, its formation and accumulation were increased by many environmental stresses (Bajji et al., 2001; Los et al., 2019; Cui et al., 2019).

Similarly, other researchers reported increased H_2_O_2_ content in tomato and wheat caused by CP treatments (Rahman et al., 2018; Adhikari et al., 2020). Under normal conditions in plant cells, Reactive Oxygen Species (ROS) is a component found in normal cellular metabolism form, especially in the mitochondria *via* enzymatic and non-enzymatic chemical reduction (Mehla et al., 2017; Raja et al., 2017; Hasanuzzaman et al., 2019) so, under these conditions, there is a balance seen between the production of ROS and its consumption in any plant. Thus, in the germination stage of seeds it was observed that H_2_O_2_, as a signaling molecule, interferes to maintain a low ABA/GA ratio, which raises α-amylase activity (Rahman et al., 2018; Adhikari et al., 2020). In these experiments, CP did not cause noticeable change at lower power (80 W) on H_2_O_2_ content, and the active production of ROS by increasing the power and exposure time of CP can not only be correlated with cellular metabolism. CP has the potential to act as a stress agent in plasma-treated seeds of *S. leriifolia* because it is a mix of reactive oxygen (ROS), nitrogen species (NOS), charged particles, excited molecules, and UV photons (Groot et al., 2018). Based on the outcomes of MDA and H_2_O_2_ accumulations, CP used at a higher power and for a prolonged exposure time induces a stressful condition (**Figures 8**, **9**). Furthermore, these results confirmed differences in the results of SEM (**Figures 2**–**5**). Correlation analysis (**Table 4**) reflected significant positive Pearson correlations between MDA and GP, and GS. However, the correlations between it, and seedling length were negative. Previous studies reported that the content of MDA in seedlings exposed to CP was lower than in the control seedlings. Despite these studies, in this experiment α-amylase and antioxidant enzymes activities increased in the same trend as increasing MDA and H_2_O_2_ content (Ling et al., 2015; Li et al., 2017; Adhikari et al., 2020). The interaction between biochemical characteristics in treated seed cells using different treatments is complex and depends on treatment characteristics, the time and route of exposure as well as the compounds of seeds and the time of characteristics measurement. In addition, the activation of amylase activity and increasing germination with increasing MDA is related to nitrogen species and compounds, more precisely nitric oxide (NO), which results from CP treatment of seeds (Miransari and Smith, 2014; Gómez-Ramírez et al., 2017)

### 3.8 Antioxidant enzyme activity [Superoxide dismutase (SOD) and catalase]

**Figure 10:**
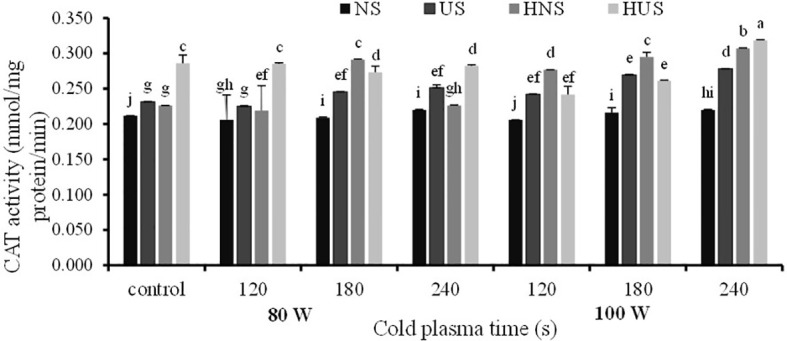
The effect of power and exposure time of cold plasma and seed situation on CAT activity of *S. leriifolia* germinated seeds. Normal seeds (NS), Uncovered seeds (US), Hydro-primed normal seeds (HNS) and Hydro-primed uncovered seeds (HUS). Values represent mean S.E. Different letters indicate significant differences at the 1% level according to the LSD test.

The statistical analysis of *S. leriifolia* germinated seeds (**Table 6**) indicated that the individual effect of SS, CP power, and exposure time of CP had a significant effect on the SOD enzyme activity. Two-way interactions between SS and CP time and power of CP and time of CP had a highly significant (p< 0.001) effect on the SOD activity (**Table 6**). The changes in SOD enzyme activity of *S. leriifolia* under the two-way interaction of power of CP and exposure time of CP and SS and exposure time of CP are presented in **Tables 2** and **3**. The results of two-way interactions between SS and exposure time of CP showed that the activity of this enzyme increased in all SS by increasing the exposure time of CP compared with the controls (free-CP treatment in all seed situations) (**Table 2**). However, the maximum activity of SOD enzyme without significant difference was recorded in the US seeds (0.243 U/mg), HNS seeds (0.246 U/mg) after 120 s, HUS seeds (0.244 U/mg) after 180 s, and NS seeds (0.243 U/mg) after 240 s (**Table 2**). The results showed that the two-way interaction of power of CP and exposure time of CP increased the activity of SOD enzyme in all treatments compared with the control (**Table 3**). Increasing the exposure duration of CP at 80 W had no significant effect on SOD enzyme activity; however, increasing the exposure time of CP at 100 W lowered the activity of this enzyme (**Table 3**). The statistical analysis (**Table 6**) indicated that the individual effect of SS, CP power and time, and two- and three-way interactions among them had a significant (*p*< 0.001) impact on the CAT enzyme activity. The effect of three-way interactions between SS, the power of CP, and exposure time of CP on CAT enzyme activity of *S. leriifolia* germinated seeds was shown in **Figure 10**. There were no significant differences in CAT enzyme activity of NS and HUS by increasing the power and exposure time of CP, separately (**Figure 10**). However, the activity of this enzyme in US and HNS increased by increasing the power and exposure time of CP, separately (**Figure 10**). Furthermore, the maximum activity of CAT enzyme was obtained in HUS (**Figure 10**).

As mentioned, ROSs are produced under normal cellular conditions, in which antioxidant enzymes, such as SOD and CAT, affect the balance of its content because they enable it to combat ROS toxicity and eliminate its harmful effects. However, the increase in antioxidant enzyme activity can be correlated with the activation of energy metabolism during seed germination, in which ROS are actively formed (Pehlivan, 2017). It is reported that SOD is one of the critical enzymatic antioxidants that reduces superoxide into H_2_O_2_, which is converted into water by CAT and various peroxidases (Ighodaro and Akinloye, 2018). On stressful conditions of the plant, e.g., extreme CP oxidative stress was reported that antioxidants activity increased and scavenged the toxic radicals and helped the plants to survive (Ling et al., 2015; Guo et al., 2017; Li et al., 2017). The effects of CP on the drastic stimulations of CAT and SOD activities in *Andrographis paniculata*, rapeseed, and *Astragalus fridae* (Tong et al., 2014; Ling et al., 2015; Moghanloo et al., 2019) were reported.

## 4 Conclusion

Overall, owing to the medicinal importance of *S. leriifolia*, finding a solution to overcome the physical and physiological dormancy of its seed and save it using environmentally friendly techniques is essential. The maximum reactive excited species under low-pressure CP in the air using 80 and 100 W was NOS. The SEM images showed that more changes (i.e., deformities and cracks) were observed in hydro-primed normal and uncovered seeds. Both uncovering and CP treatments showed enhanced α-amylase activity, which is the enzyme mainly responsible for germination and enhancing radicle growth. Nevertheless, as observed in this plant’s response to uncovering, hydro-primed, and CP treatments, it seems that the phenomenon of dormancy in this plant is complex. The remove of physical dormancy via removing the cover of seeds could not enhance the germination percentage and rate as well as SVI and SVII in most of the treatments by low power and shorter exposure time of CP. However, the effect of this plasma on the seeds of *S. leriifolia* plant, which contain high-fat and protein contents and have hard seed coats, demonstrated that 100 W of CP for 240 s as the crucial and optimal enhancement conditions. This treatment could increase seed germination percentage by more than twice that seen in the control. So, further research is recommended using other types of CP, as a harmless solution, to improve germination and overcome seed dormancy of *S. leriifolia* species by studying more enzymes and their gene expression, such as amylase, lipase, proteinase activity, and antioxidant enzyme activity.

## Data availability statement

The original contributions presented in the study are included in the article/supplementary material. Further inquiries can be directed to the corresponding author.

## Author contributions

SG contributed to CP experiments on seeds, SEM image preparation, ROS enzyme activity data extraction, carried out all data of this manuscript analysis, and prepared the figures. HM conceived and designed research, supervised the whole experiment, and co-wrote the manuscript. ZG designed research and co-wrote the manuscript. MG revised the manuscript. All authors read and approved the final manuscript.

## References

[B1] Adhikari B. Adhikari M. Ghimire B. Adhikari B. Park G. Choi E. H. (2020). Cold plasma seed priming modulates growth, redox homeostasis and stress response by inducing reactive species in tomato (*Solanum lycopersicum*). Free Radical Biol. Med. 156, 57–69. doi: 10.1016/j.freeradbiomed.2020.06.003 32561321

[B2] Amini M. Kafi M. Parsa M. (2018). Evaluation of the effects of various fertilizers (N, p, K) application on morphological and growth characteristics of *Salvia leriifolia* benth. Sci. Rep. 8, 11655. doi: 10.1038/s41598-018-30200-7 30076394 PMC6076249

[B3] Bajji M. Lutts S. Kinet J. (2001). Water deficit effects on solute contribution to osmotic adjustment as a function of leaf ageing in three durum wheat (*Triticum durum* desf.) cultivars performing differently in arid conditions. Plant Sciences,160 4, 669–681. doi: 10.1016/S0168-9452(00)00443-X 11448742

[B4] Baskin C. C. Baskin J. M. (2014). Seeds: ecology, biogeography, and, evolution of dormancy and germination (Lexington, KY, USA: Academic Press), pp, 101–116. doi: 10.1016/C2013-0-00597-X

[B5] Beers R. F. Sizer I. W. (1952). Colorimetric method for estimation of catalase. J. Biol. Chem. 195, 133–139. doi: 10.1016/j.plaphy.2013.11.016 14938361

[B6] Bormashenko E. Grynyov R. Bormashenko Y. Drori E. (2012). Cold radio frequency plasma treatment modifies wettability and germination speed of plant seeds. Sci. Reports 2 1, 741. doi: 10.1038/srep00741 PMC3473364 23077725

[B7] Bormashenko E. Shapira Y. Grynyov R. Bormashenko Y. Drori E. (2015). Interaction of cold radiofrequency plasma with seeds of beans (*Phaseolus vulgaris*). J. Exp. Bot. 66, 4013–4021. doi: 10.1093/jxb/erv206 25948708 PMC4473997

[B8] Cui D. Yin Y. Wang J. Wang Z. Ding H. Ma R.. (2019). Research on the physiobiochemical mechanism of non-thermal plasma-regulated seed germination and early seedling development in arabidopsis. Front. Plant Sci. 10. doi: 10.3389/fpls.2019.01322 PMC6857620 31781132

[B9] Damalas C. A. Koutroubas S. D. Fotiadis S. (2019). Hydro-priming effects on seed germination and field performance of faba bean in spring sowing. Agriculture 9, 201. doi: 10.3390/agriculture9090201

[B10] Dhayal M. Lee S. Y. Park S. U. (2006). Using low-pressure plasma for *Carthamus tinctorium* l. seed surface modification. Vacuum J. 80, 499–506. doi: 10.1016/j.vacuum.2005.06.008

[B11] Dobrynin D. Fridman G. Friedman G. Fridman A. (2009). Physical and biological mechanisms of direct plasma interaction with living tissue. New J. Physics. 1, 2–26. doi: 10.1088/1367-2630/11/11/115020

[B12] Filatova I. I. Azharonok V. V. Goncharik S. V. Lushkevich V. A. Zhukovsky A. G. Gadzhieva G. I. (2014). Effect of RF plasma treatment on the germination and phytosanitary state of seeds. Appl. Spectrosc. 81, 250–256. doi: 10.1007/s10812-014-9918-5

[B13] Filatova I. Lyushkevich V. Goncharik S. Zhukovsky A. Krupenko N. Kalatskaja J. (2020). The effect of low-pressure plasma treatment of seeds on the plant resistance to pathogens and crop yields. J. Phys. D: Appl. Phys. 53, 244001. doi: 10.1088/1361-6463/ab7960

[B14] Finch-Savage W. E. Leubner-Metzger G. (2006). Seed dormancy and the control of germination. New Phytol. 171, 3, 501–523. doi: 10.1111/j.1469-8137.2006.01787.x 16866955

[B15] Finkelstein R. R. (2010). The role of hormones during seed development and germination. Ed. Davies P. J. (Dordrecht: Springer), 549–573.

[B16] Geneve R. L. (2003). Impact of temperature on seed dormancy. Hortic. Sci. 38, 3, 336–341. doi: 10.21273/HORTSCI.38.3.336

[B17] Ghassemi-Golezani K. Yaghoubian I. Raey Y. (2016). The impact of hydro-priming duration on seed invigoration and field emergence of milk thistle. J. Biodiversity Environ. Sci. 9, 229–234.

[B18] Gilani M. M. Tigabu M. Liu B. Farooq T. H. Rashid M. H. U. Ramazan M.. (2021). Seed germination and seedling emergence of four tree species of southern China in response to acid rain. J. For. Rese 32 (2), 471–481. doi: 10.1007/s11676-020-01102-0

[B19] Gómez-Ramírez A. López-Santos C. Cantos M. García J. L. Molina R. Cotrino J.. (2017). Surface chemistry and germination improvement of quinoa seeds subjected to plasma activation. Sci. Rep. 7, 12. 5924. doi: 10.1038/s41598-017-06164-5 28725039 PMC5517418

[B20] Groot G. J. J. B. Hundt A. Murphy A. B. Bange M. P. Mai-Prochnow A. (2018). Cold plasma treatment for cotton seed germination improvement. Sci. Rep. 8, 14372. doi: 10.1038/s41598-018-32692-9 30258075 PMC6158256

[B21] Guo Q. Wang Y. Zhang H. Qu G. Wang T. Sun Q.. (2017). Alleviation of adverse effects of drought stress on wheat seed germination using atmospheric dielectric barrier discharge plasma treatment. Sci. Rep. 7, 1–14. doi: 10.1038/s41598-017-16944-8 29192193 PMC5709406

[B22] Hasanuzzaman M. Bhuyan M. Anee T. I. Parvin K. Nahar K. Mahmud J. A.. (2019). Regulation of ascorbate-glutathione pathway in mitigating oxidative damage in plants under abiotic stress. Antioxidants 8, 384. doi: 10.3390/antiox8090384 31505852 PMC6770940

[B23] Hatami M. Hadian J. Ghorbanpour M. (2017). Mechanisms underlying toxicity and stimulatory role of single-walled carbon nanotubes in *Hyoscyamus niger* during drought stress simulated by polyethylene glycol. J. Hazardous Materials. 324, 306–320. doi: 10.1016/j.jhazmat.2016.10.064 27810325

[B24] Heath R. L. Packer L. (1968). Photoperoxidation in isolated chloroplast. i. kinetics and stoichiometry of fatty acid peroxidation. Biochem. biophysics Rep. 125, 189–198. doi: 10.1016/0003-9861(68)90654-1 5655425

[B25] Hoppanová L. Medvecká V. Dylíková J. Hudecová D. B. Kryštofová S. Zahoranová A. (2020). Low-temperature plasma applications in chemical fungicide treatment reduction. Acta chimica slovenica 13, 26–33. doi: 10.2478/acs-2020-0005

[B26] Hussain S. Zheng M. Khan F. Khaliq A. Fahad S. Peng S.. (2015). Benefits of rice seed priming are offset permanently by prolonged storage and the storage conditions. Sci. Rep. 5, 8101. doi: 10.1038/srep08101 25631923 PMC4309961

[B27] Ighodaro O. M. Akinloye O. A. (2018). First line defense antioxidants-superoxide dismutase (SOD), catalase (CAT) and glutathione peroxidase (GPX): Their fundamental role in the entire antioxidant defence grid. Alexandria J. Med. 54, 287–293. doi: 10.1016/j.ajme.2017.09.001

[B28] Illangakoon T. K. Ella E. S. Ismail A. M. Marambe B. Keerthisena R. S. K. Bentota A. P.. (2016). Impact of variety and seed priming on anaerobic germination tolerance of rice (*Oryza sativa* l.) varieties in Sri Lanka. Trop. Agric. Res. 28, 26–37. doi: 10.4038/tar.v28i1.8181

[B29] Iranbakhsh A. Ardebili N. O. Ardebili Z. O. Shafaati M. Ghoranneviss M. (2018). Non-thermal plasma induced expression of heat shock factor A4A and improved wheat (*Triticum aestivum* l.) growth and resistance against salt stress. Plasma Chem. Plasma Process. 38, 29–44. doi: 10.1007/s11090-017-9861-3

[B30] Jiang J. He X. Li L. Li J. Shao H. Xu Q.. (2014). Effect of cold plasma treatment on seed germination and growth of wheat. Plasma Sci. Technol. 16, 54–57. doi: 10.1088/1009-0630/16/1/12

[B31] Ji S. H. Choi K. H. Pengkit A. Im J. S. Kim J. S. Kim Y. H.. (2016). Effects of high voltage nanosecond pulsed plasma and micro DBD plasma on seed germination, growth development and physiological activities in spinach. Arch. Biochem. Biophysics 605, 117–128. doi: 10.1016/j.abb.2016.02.028 26944552

[B32] Jones R. L. Varner J. E. (1967). The bioassay of gibberellins. Planta 72, 155–161. doi: 10.2307/23366568 24554208

[B33] Junglee S. Laurent U. Sallanon H. Felicie L. (2014). Optimized assay for hydrogen peroxide determination in plant tissue using potassium iodide. Am. J. Analytical Chem. 5, 730–736. doi: 10.4236/ajac.2014.511081

[B34] Kordas L. Pusz W. Czapka T. Kacprzyk R. (2015). Effect of low-temperature plasma on fungus colonization of winter wheat grain and seed quality. Polish J. Environ. Stud. 24, 433–438.

[B35] Laroussi M. Leipold F. (2004). Evaluation of the roles of reactive species, heat, and UV radiation in the inactivation of bacterial cells by air plasmas at atmospheric pressure. Int. J. Mass Spectrometry 233, 81–86. doi: 10.1016/j.ijms.2003.11.016

[B36] Lee E.-J. Khan M. S. I. Shim J. Kim Y.-J. (2018). Roles of oxides of nitrogen on quality enhancement of soybean sprout during hydroponic production using plasma discharged water recycling technology. Sci. Rep. 8, 16872. doi: 10.1038/s41598-018-35385-5 30443039 PMC6237935

[B37] Le T. Q. X. Nguyen L. N. Nguyen T. T. Choi E. H. Nguyen Q. L. Kaushik N. K.. (2022). Effects of cold plasma treatment on physical modification and endogenous hormone regulation in enhancing seed germination and radicle growth of mung bean. Appl. Sci. 12 (20), 10308. doi: 10.3390/app122010308

[B38] Li W. Chen G. Fang Y. Wang T. Wu Y. Wu Y.. (2021). Hydrogen peroxide as a systemic messenger in the photosynthetic induction of mulberry leaves. J. For. Res. 32, 945–952. doi: 10.1007/s11676-020-01165-z

[B39] Ling L. Jiafeng J. Jiangang L. Minchong S. Xin H. Hanliang S.. (2014). Effects of cold plasma treatment on seed germination and seedling growth of soybean. Sci. Rep. 4 (1), 5859. doi: 10.1038/srep05859 25080862 PMC4118186

[B40] Ling L. Jiangang L. Minchong S. H. Chunlei Z. Yuanhua D. (2015). Cold plasma treatment enhances oilseed rape seed germination under drought stress. Sci. Rep. 5 (1), 13033. doi: 10.1038/srep13033 26264651 PMC4533018

[B41] Li Y. Wang T. Meng Y. Qu G. Sun Q. Liang D.. (2017). Air atmospheric dielectric barrier discharge plasma induced germination and growth enhancement of wheat seed. Plasma Chem. Plasma Processing. 37, 1621–1634. doi: 10.1007/s11090-017-9835-5

[B42] Loizzo M. R. Menichini F. Tundis R. Bonesi M. Conforti F. Nadjafi F.. (2010). *Salvia leriifolia* benth (Lamiaceae) extract demonstrates *in vitro* antioxidant properties and cholinesterase inhibitory activity. Nutr. Res. 30, 823. doi: 10.5650/jos.58.443 21147365

[B43] Los A. Ziuzina. D. Boehm D. Cullen P. J. Bourke P. (2019). Investigation of mechanisms involved in germination enhancement of wheat (*Triticum aestivum*) by cold plasma: effects on seed surface chemistry and characteristics. Plasma Processes Polymers 16, 1800148. doi: 10.1002/ppap.201800148

[B44] Machado-Moreira B. Tiwari B. K. Richards K. G. Abram F. Burgess C. M. (2021). Application of plasma activated water for decontamination of alfalfa and mung bean seeds. Food Microbiol. 96, 103708. doi: 10.1016/j.fm.2020.103708 33494890

[B45] Maguire J. D. (1962). Speed of germination-aid in selection and evaluation for seedling emergence and vigor. Crop Sci. 2, 176–177. doi: 10.2135/cropsci1962.0011183X000200020033x

[B46] Magureanu M. Sîrbu R. Dobrin D. Gîdea M. (2018). Stimulation of the germination and early growth of tomato seeds by non-thermal plasma. Plasma Chem. Plasma Process. 38, 989–1001. doi: 10.1007/s11090-018-9916-0

[B47] Mehla N. Sindhi V. Josula D. Bisht P. Wani S. H. (2017). “An introduction to antioxidants and their roles in plant stress tolerance,” in Reactive oxygen species and antioxidant systems in plants: Role and regulation under abiotic stress. Eds. Khan M. I. R. Khan N. A. (Singapore: Springer), 1–23.

[B48] Mildaziene V. Aleknaviciute V. Zukiene R. Pauzaite G. Nauciene Z. Filatova I.. (2019). Treatment of common sunflower (*Helianthus annus* l.) seeds with radio-frequency electromagnetic field and cold plasma induces changes in seed phytohormone balance, seedling development and leaf protein expression. Sci. Rep. 9, 6437. doi: 10.1038/s41598-019-42893-5 31015543 PMC6478675

[B49] Miransari M. Smith D. L. (2014). Plant hormones and seed germination. Environ. Exp. Botany. 99, 110–121. doi: 10.1016/j.envexpbot.2013.11.005

[B50] Misra H. P. Fridovich I. (1972). The role of superoxide anion in the autoxidation of epinephrine and simple assay for superoxide dismutase. J. Biol. Chem. 244, 6049–6055. doi: 10.1016/S0021-9258(19)45228-9 4623845

[B51] Misra N. Kaur S. Tiwari B. K. Kaur A. Singh N. Cullen P. J. (2015). Atmospheric pressure cold plasma (ACP) treatment of wheat flour. Food Hydrocolloids. 44, 115–121. doi: 10.1016/j.foodhyd.2014.08.019

[B52] Modarres M. Asili J. Lahouti M. Iranshahi M. Sahebkar A. (2014). Simultaneous determination of rosmarinic acid, salvianolic acid b and caffeic acid in *Salvia leriifolia* benth. root, leaf and callus extracts using a high-performance liquid chromatography with diode-array detection technique. J. Liquid Chromatogr. Related Technologies. 37, 1721–1730. doi: 10.1080/10826076.2013.807466

[B53] Moghanloo M. Iranbakhsh A. R. Ebadi M. Oraghi Ardebili Z. (2019). Differential physiology and expression of phenylalanine ammonia lyase (PAL) and universal stress protein (USP) in the endangered species *Astragalus fridae* following seed priming with cold plasma and manipulation of culture medium with silica nanoparticles. 3Biotech. 9 7, 288. doi: 10.1007/s13205-019-1822-5 PMC6597672 31297304

[B54] Nishime T. M. C. Wannicke N. Horn S. Weltmann K. D. Brust H. (2020). A coaxial dielectric barrier discharge reactor for treatment of winter wheat seeds. Appl. Sci. 10, 7133. doi: 10.3390/app10207133

[B55] Pang L. Xiao J. Ma J. Yan L. (2021). Hyperspectral imaging technology to detect the vigor of thermal-damaged quercus variabilis seeds. J. For. Res. 32, 461–469. doi: 10.1007/s11676-020-01144-4

[B56] Park Y. Oh K. S. Oh J. Seok D. C. Kim S. B. Yoo S. J.. (2018). The biological effects of surface dielectric barrier discharge on seed germination and plant growth with barley. Plasma Processes Polymers 15, 1600056. doi: 10.1002/PPAP.201600056

[B57] Pehlivan F. E. (2017). “Free radicals and antioxidant system in seed biology,” in Advances in seed biology (London, UK: InTech), 167.

[B58] Pereira J. W. L. Filho P. A. M. Albuquerque M. B. Nogueira R. J. M. C. Santos R. C. (2012). Biochemical changes in peanut genotypes submitted to moderate water stress. Rev. Ciec. Agron. 43, 766–773. doi: 10.1590/S1806-66902012000400019

[B59] Pour H. Nemati S. H. Tehranifar A. Shoor M. Joharchi M.R. (2010). Evaluation of seed arrangement, and culture depth on germination characteristics and seedling establishment in order to domestication of nowruzak (*Salvia leriifolia* benth). J. Hortic. Sci. 24, 136–141. doi: 10.22067/jhorts4.v1389i2.7986

[B60] Priatama R. A. Pervitasari A. N. Park S. Park S. J. Lee Y. K. (2022). Current advancements in the molecular mechanism of plasma treatment for seed germination and plant growth. Int. J. Mol. Sci. 23, 4609. doi: 10.3390/ijms23094609 35562997 PMC9105374

[B61] Rahman M. M. Sajib S. A. Rahi M. S. Tahura S. Roy N. C. Parvez S.. (2018). Mechanisms and signaling associated with LPDBD plasma mediated growth improvement in wheat. Sci. Rep. 8, 10498. doi: 10.1038/s41598-018-28960-3 30002439 PMC6043519

[B62] Rajagopalan G. Krishnan C. (2008). Immobilization of malto-oligosaccharide forming α-amylase from bacillus subtilis KCC103: Properties and application in starch hydrolysis. J. Chem. Technol. Biotechnol. 83, 1511–1517. doi: 10.1002/jctb.1922

[B63] Raja V. Majeed U. Kang H. Andrabi K. I. John R. (2017). Abiotic stress: Interplay between ROS, hormones and MAPKs. Environ. Exp. Bot. 137, 142–157. doi: 10.1016/j.envexpbot.2017.02.010

[B64] Rechinger K. H. (1982). Flora iranica. N.150, academishe Druk.u.Verlagsustalt gratz 439.

[B65] Saberi M. Modarres-Sanavy S. A. M. Zare R. Ghomi H. (2018). Amelioration of photosynthesis and quality of wheat under nonthermal radio frequency plasma treatment. Sci. Rep. 8, 11655. doi: 10.1038/s41598-018-30200-7 30076394 PMC6076249

[B66] Sadhu S. Thirumdas R. Deshmukh R. R. Annapure U. S. (2017). Influence of cold plasma on the enzymatic activity in germinating mung beans (*Vigna radiate*). Food Sci. Technol. 78, 97–104. doi: 10.1016/j.lwt.2016.12.026

[B67] Sajib S. A. Billah M. Mahmud S. Miah M. Hossain F. Omar F. B.. (2020). Plasma activated water: the next generation ecofriendly stimulant for enhancing plant seed germination, vigor and increased enzyme activity, a study on black gram (*Vigna mungo* l.). Plasma Chem. Plasma Process. 40, 119–143. doi: 10.1007/s11090-019-10028-3

[B68] Savelev S. U. Okello E. J. Perry E. K. (2004). Butyryl- and acetyl-cholinesterase inhibitory activities in essential oils of salvia species and their constituents. Phytother Res. 18, 315–324. doi: 10.1002/ptr.1451 15162368

[B69] Selwyn G. S. Herrmann H. W. Park J. Henins I. (2001). Materials processing using an atmospheric pressure, RF-generated plasma source. Contributions to Plasma Phys. 6, 610–619. doi: 10.1002/1521-3986(200111)41:6<610

[B70] Sera B. Gajdova I. Cernak M. Gavril B. Hnatiuce E. Kovacik D.. (2012). How various plasma sources may affect seed germination and growth? IEEE Plasma Sci. 39, 1365–1369. doi: 10.1109/OPTIM.2012.6231880

[B71] Sera B. Spatenka P. Sery M. Vrchotova N. Hruskova I. (2010). Influence of plasma treatment on wheat and oat germination and early growth. IEEE Trans. Plasma Sci. 38, 2963–2967. doi: 10.1109/TPS.2010.2060728

[B72] Serry F. S. Ghamari-Zare A. Shahrzaad S. NaderiShahab M. A. Kalate-jary S. (2012). Effect of physic-chemical treatments on seed germination of *Salvia leriifolia* benth. Iranian J. Medicinal Aromatic Plants 27 (4), 659–667. doi: 10.22092/IJMAPR.2012.4515

[B73] Shelar A. Singh A. V. Dietrich P. Maharjan R. S. Thissen A. Didwal P.. (2022). Emerging cold plasma treatment and machine learning prospects for seed priming: a step towards sustainable food production. R. Soc. Chem. 12, 10467–10488. doi: 10.1039/D2RA00809B PMC8982346 35425017

[B74] Sivachandiran L. Khacef A. (2017). Enhanced seed germination and plant growth by atmospheric pressure cold air plasma: combined effect of seed and water treatment. RSC Advances. 7, 1822–1832. doi: 10.1039/C6RA24762H

[B75] Soltania A. Gholipoor M. Zeinalia E. (2006). Seed reserve utilization and seedling growth of wheat as affected by drought and salinity. Environ. Exp. Bot. 55, 195–200. doi: 10.1016/j.envexpbot.2004.10.012

[B76] Song J. S. Lee M. J. Ra J. E. Lee K. S. Eom S. Ham H. M.. (2020). Growth and bioactive phytochemicals in barley (*Hordeum vulgare* l.) sprouts affected by atmospheric pressure plasma during seed germination. J. Phys. D: Appl. Phys. 53, 314002. doi: 10.1088/1361-6463/ab810d

[B77] Srisonphan S. (2018). Tuning surface wettability through hot carrier-initiated impact ionization in cold plasma. ACS Appl. Materials Interfaces 10, 11297–11304. doi: 10.1021/acsami.7b19495 29547259

[B78] Stabell S. Upadhyaya M. K. Ellis B. E. (1998). Role of seed coat in regulation of seed dormancy in houndstongue (*Cynoglossum officinale*). Weed Sci. 46 (3), 344–350. doi: 10.1017/S0043174500089529

[B79] Stola´rik T. Henselova M. Martinka M. Nova´k O. Zahoranová A. Cerna´k M. (2015). Effect of low-temperature plasma on the structure of seeds, growth and metabolism of endogenous phytohormones in pea (*Pisum sativum* l.). Plasma Chem. Plasma Process. 35, 659–676. doi: 10.1007/s11090-015-9627-8

[B80] Sun T. P. (2008). Gibberellin metabolism, perception and signaling pathways in arabidopsis. Plant Biol. 6, 103–131. doi: 10.1199/tab.0103 PMC3243332 22303234

[B81] Sun J. Wu D. Xu J. Rasmussen S. K. Shu X. (2015). Characterisation of starch during germination and seedling development of a rice mutant with a high content of resistant starch. J. Cereal Sci. 62, 94–101. doi: 10.1016/j.jcs.2015.01.002

[B82] Tezuka T. Yokoyama H. Tanaka H. Shiozaki S. Oda M. (2012). Seed and embryo germination in *Ardisia crenata* . J. Bot. 2012, 1–10. doi: 10.1155/2012/679765

[B83] Thirumdas R. Trimukhe A. Deshmukh R. R. Annapure U. S. (2017). Functional and rheological properties of cold plasma treated rice starch. Carbohydr. Polymers 157, 1723–1731. doi: 10.1016/j.carbpol.2016.11.050 27987888

[B84] Tong J. Y. Zhang X. L. Zhan R. T. Chan W. W. Yang S. Z. (2014). Effect of atmospheric pressure air plasma pretreatment on the seed germination and early growth of *Andragraphis paniculate* . Plasma Sci. Tech. 16, 260–266. doi: 10.1088/1009-0630/16/3/16

[B85] Wang X. Q. Zhou R. W. Bazaka K. Murphy A. B. Ostrikov K. (2017). Spectral characteristics of cotton seeds treated by a dielectric barrier discharge plasma. Sci. Rep. 7, 5601. doi: 10.1038/s41598-017-04963-4 28717249 PMC5514119

[B86] Zahoranová A. Maria H. Hudecová D. Barbora K. Dusan K. Veronika M.. (2016). Effect of cold atmospheric pressure plasma on the wheat seedlings vigor and on the inactivation of microorganisms on the seeds surface. Plasma Chem. Plasma Process. 36, 397–414. doi: 10.1007/s11090-015-9684-z

[B87] Zulfiqar F. (2021). Effect of seed priming on horticultural crops. Scientia Horticult 286, 110197. doi: 10.1016/j.scienta.2021.110197

[B88] Zulfiqar F. Chen J. Finnegan P. M. Younis A. Nafees M. Zorrig W.. (2021). Application of trehalose and salicylic acid mitigates drought stress in sweet basil and improves plant growth. Plants 10, 1078. doi: 10.3390/plants10061078 34072096 PMC8230182

[B89] Zulfiqar F. Nafees M. Chen J. Darras A. Ferrante A. Hancock J. T.. (2022). Chemical priming inhances plant tolerance to salt stress. Front. Plant Sci. 13. doi: 10.3389/fpls.2022.94692 PMC9490053 36160964

